# Epithelial immune regulation of inflammatory airway diseases: Chronic rhinosinusitis with nasal polyps (CRSwNP) 

**DOI:** 10.5414/ALX02296E

**Published:** 2022-04-29

**Authors:** Ludger Klimek, Jan Hagemann, Hans-Jürgen Welkoborsky, Mandy Cuevas, Ingrid Casper, Ulrike Förster-Ruhrmann, Felix Klimek, Constantin A. Hintschich, Tilman Huppertz, Christoph Bergmann, Peter-Valentin Tomazic, Sven Becker

**Affiliations:** 1 Center for Rhinology and Allergology, Wiesbaden,; 2 Clinic and Polyclinic for Otolaryngology, University Medical Center Mainz, Mainz,; 3 Clinic for Ear, Nose and Throat Medicine, Head and Neck Surgery, Nordstadt Clinic of the KRH, Hannover,; 4 Clinic and Polyclinic for Otolaryngology, University Hospital Carl Gustav Carus, TU Dresden, Dresden,; 5 HNO-University Clinic Charité, Berlin,; 6 Clinic and Polyclinic for Ear, Nose and Throat Medicine, University Hospital Regensburg, Regensburg,; 7 HNO RKM740 Interdisciplinary Specialist Clinic, Düsseldorf, Germany,; 8 HNO-University Clinic Graz, Medical University Graz, Austria, and; 9 HNO-University Clinic Tübingen, Germany

**Keywords:** alarmin, CRS, CRSwNP, epithelial immune regulation, T2-inflammation, Tezepelumab, TSLP

## Abstract

Background: The epithelial immune regulation is an essential and protective feature of the barrier function of the mucous membranes of the airways. Damage to the epithelial barrier can result in chronic inflammatory diseases, such as chronic rhinosinusitis (CRS) or bronchial asthma. Thymic stromal lymphopoietin (TSLP) is a central regulator in the epithelial barrier function and is associated with type 2 (T2) and non-T2 inflammation. Materials and methods: The immunology of chronic rhinosinusitis with polyposis nasi (CRSwNP) was analyzed in a literature search, and the existing evidence was determined through searches in Medline, Pubmed as well as the national and international study and guideline registers and the Cochrane Library. Human studies or studies on human cells that were published between 2010 and 2020 and in which the immune mechanisms of TSLP in T2 and non-T2 inflammation were examined were considered. Results: TSLP is an epithelial cytokine (alarmin) and a central regulator of the immune reaction, especially in the case of chronic airway inflammation. Induction of TSLP is implicated in the pathogenesis of many diseases like CRS and triggers a cascade of subsequent inflammatory reactions. Conclusion: Treatment with TSLP-blocking monoclonal antibodies could therefore open up interesting therapeutic options. The long-term safety and effectiveness of TSLP blockade has yet to be investigated.

## Introduction 

An intact mucosal barrier is crucial for the maintenance of tissue homeostasis as it protects the organism from infections, environmental toxins, pollutants, and allergens [1]. A disrupted epithelial barrier has been demonstrated in allergic and autoimmune diseases such as allergic rhinitis and chronic rhinosinusitis (CRS), but also in similar diseases such as atopic dermatitis, asthma, eosinophilic esophagitis, celiac disease, and inflammatory bowel disease [1]. 

Some authors even suggest that the increase in epithelial barrier-damaging agents associated with industrialization, urbanization, and the associated “modern life” underlies the rise in allergic, autoimmune, and other chronic mucosal diseases [1]. 

It seems to be proven that, in addition to the epithelial barrier, an intact mucosal immune system is a prerequisite for the prevention of chronic inflammatory mucosal diseases, and mucosal immune dysfunction is also involved in the development of diseases such as allergic rhinitis and CRS [1, 2, 3, 4]. 

Thymic stromal lymphopoietin (TSLP) is a cytokine primarily expressed by airway epithelium and released in response to environmental factors, triggering various inflammatory processes [5]. 

TSLP expression is increased in the airways of asthma patients compared to healthy individuals and correlates with disease severity and lung function. Polymorphisms in the *TSLP* gene have been associated with asthma. 

Evidence suggests that TSLP is an important factor in the pathophysiology of chronic inflammatory airway diseases, promoting eosinophilic (allergic and non-allergic) inflammation, non-eosinophilic inflammation, and airway structural changes through its effects on a variety of adaptive and innate immune cells and epithelial cells [5]. 

Clinical trials of TSLP blockade with monoclonal antibodies in patients with chronic inflammatory airway disease have been highly successful [5]. 

## Chronic rhinosinusitis with polyposis nasi and bronchial asthma 

CRS is an inflammatory disease of the mucous membranes of the nose and sinuses [6, 7, 8, 9]. Globally, CRS affects ~ 5 – 12% of the general population, and the cost to healthcare systems and national economies is substantial [6, 7, 8, 10]. CRS is divided into a phenotype with (CRSwNP) and one without the development of nasal polyps (CRSsNP) [9, 10, 11, 12]. CRSwNP is also referred to as polyposis nasi et sinuum and is associated with endoscopic and/or radiologic evidence of polypoid hyperplastic tissue in the nasal cavity and/or paranasal sinuses. CRSwNP is an immunologically triggered chronic inflammatory disease of the mucosa and subepithelial tissues for which specific endotype-based immunologic therapies have been developed only in the last decade [4, 13, 14, 15, 16], whereas immunotherapies [17, 18, 19, 20, 21] or avoidance measures [22, 23] for exogenous allergic nasal mucosal diseases have been a therapeutic standard for a long time. 

The inflammation associated with CRS is heterogeneous and has been associated with different inflammatory endotypes. Among the most common endotypes is type 2 (T2) inflammation [15]. As in asthma, the majority of CRS patients in Europe have a T2 endotype of inflammation [24, 25, 26, 27, 28, 29, 30, 31, 32]. It is due to this similarity to the pathophysiology of asthma that most of the biologics approved for asthma therapy have also been shown to be effective in CRSwNP [4, 15, 28, 33]. 

Alternatively, non-T2 inflammation may be present (for example, neutrophilic) [34, 35, 36]. In patients with severe respiratory disease that cannot be adequately controlled by inhaled therapies, knowledge of the patient’s inflammatory endotype(s) helps to select the optimal biologics therapy [32, 37, 38]. 

Immunoglobulin (Ig) E, eosinophils in sputum and blood, interleukin (IL)-4, IL-5, and IL-13 are considered indicative biomarkers. Because there may be evidence of multiple upregulated inflammatory pathways in each individual patient, it may be difficult to identify a single predominant endotype in individual cases [4]. Five biologics have been approved for patients with moderate-to-severe allergic and/or eosinophilic asthma, and all have shown greater efficacy in patients with T2 inflammation than in patients without T2 inflammation [39, 40, 41, 42, 43]. Currently, there are no approved biologics for non-T2 inflammation. 

The immunologic significance of the epithelial cytokine TSLP offers the possibility for a new approach to the treatment of chronic airway inflammation. Epithelial cytokines are commonly referred to as alarmins, which include IL-25 and IL-33. TSLP is released by airway epithelial cells in response to various environmental agents, such as viral and bacterial infections, in response to allergens and chemical irritants, and injury [44, 45]. Functionally, TSLP is a key substance in the initiation of immune response to environmental agents, which initiates a series of downstream inflammatory processes [5]. While TSLP initiates a distinct T2 inflammatory response [46, 47, 48], there is increasing evidence for TSLP involvement in non-T2 processes involving interactions with both immune cells and epithelial cells. The considerable extent of TSLP-mediated effects is illustrated by the broad spectrum of cell types expressing the TSLP receptor (TSLPR): eosinophils, basophils, mast cells, airway smooth muscle cells (ASMCs), innate lymphoid cells group 2 (ILC2s), lymphocytes, dendritic cells, hematopoietic progenitor cells, and monocytes/macrophages [49, 50]. In addition, TSLP appears to act as a mediator between different immune cell types and epithelial cells in the airways [5]. TSLP production seem to be increased in chronic inflammatory diseases of the airways (asthma, CRS) and skin (atopic dermatitis) [5]. TSLP expression is significantly increased in asthma patients compared to healthy individuals in the inner and outer epithelial layers of airway biopsies [51, 52, 53, 54, 55, 56, 57, 58] as well as in serum samples [59, 60], sputum [61], exhaled breath condensate [62], and bronchoalveolar lavage fluid [57, 63]. Moreover, TSLP expression has been shown to correlate with airway obstruction and disease severity in asthma patients [56, 58, 61, 63, 64]. Several elements of the pathophysiology of chronic inflammatory airway diseases, including airway hyperresponsiveness, hypersecretion, and airway remodeling, are thought to be controlled, at least in part, by the proinflammatory actions of TSLP involving cytokines such as IL-4, IL-5, and IL-13 [65]. At least in asthma, the role of TSLP is further underscored by genome-wide association studies that have found associations between asthma risk and single nucleotide polymorphisms (SNPs) in the *TSLP* gene [66, 67, 68]. Interestingly, this includes rs1837253 [69, 70], which regulates TSLP production in nasal epithelial cells [71] and is significantly positively correlated with the manifestation of asthma in CRS patients [72]. TSLP has also been associated with N-ERD (nonsteroidal anti-inflammatory drug-exacerbated respiratory disease) syndrome, also known as aspirin-exacerbated respiratory disease (AERD) or aspirin intolerance syndrome (AIS) in Europe. N-ERD is characterized by the triad of bronchial asthma, CRSwNP, and intolerance to aspirin or other nonsteroidal anti-inflammatory drugs (cyclooxygenase-1 inhibitors). Examination of nasal polyp tissue from individuals with N-ERD/AERD and those with CRS without N-ERD/AERD showed that TSLP mRNA expression was significantly increased in N-ERD/AERD [73, 74]. 

The role of TSLP in the pathogenesis of chronic inflammatory airway disease has led to the development of anti-TSLP monoclonal antibodies as a therapeutic option for these patients. In asthma, the results of clinical trials of anti-TSLP therapy are very convincing [75]. 

## Materials and methods 

For the present publication, a selective literature search was performed in Medline, Pubmed, and the national and international trials and guidelines registries and the Cochrane Library, and on the World Wide Web. Human studies or human cell studies published from 2010 to 2020 that investigated the immune mechanisms of TSLP in T2 and non-T2 inflammation were considered. In addition, current publications in literature databases of available German-language journals were analyzed. This literature search considered original and review papers in German or English language. The aim of this review is to summarize the available data on the mechanisms of action of TSLP in CRS across the spectrum of inflammatory endotypes to illustrate the therapeutic potential of novel TSLP-blocking therapies. 

Deliberately, only human studies were considered here because although the biology of the TSLP pathway appears to be similar in humans and rodents, the ability to use rodent models is limited by the generally poor transferability of rodent models to complex, heterogeneous human diseases [76, 77]. 

Search terms used were TSLP OR thymic stromal lymphopoietin AND asthma* or AND CRS* or AND CRSwNP*, using the species filter “Humans”. Review articles were also considered. The results of these searches were reviewed for relevance, that is, whether they contained information on sites of TSLP expression, TSLP effector cells, or physiological or clinical effects of TSLP, and were supplemented with other relevant articles known to the authors. 

## Immunological mechanisms of action of TSLP in T2 inflammation 

Several local effector cells play a role in the development of T2 inflammation. The interaction between airway epithelium and these cells is an important process driving eosinophilic inflammation. TSLP acts directly on immune cells involved in T2 inflammatory processes in CRS. 

### TSLP and eosinophils 

Eosinophilic inflammation contributes significantly to the physiological changes and airway remodeling in chronic inflammatory airway disease. Eosinophils are present in airways altered due to T2 inflammation but also in T2 inflammation of the skin (e.g., atopic dermatitis) and are subsequently locally activated [78, 79, 80, 81, 82, 83, 84]. 

In uncontrolled [85] or severe [86] asthma, there are locally increased eosinophils, while they may decrease in controlled asthma [87]. 

Few studies have examined the direct effect of TSLP on mature human eosinophils, and cross-sectional comparisons with cells from asthma patients compared with healthy controls are also lacking. Human eosinophils express both TSLPR and IL-7Rα subunits, and their expression is enhanced by tumor necrosis factor (TNF)-α and IL-3 [88, 89]. TSLP promotes eosinophil viability by attenuating apoptosis and induces significant production of IL-6, an eosinophil-derived neurotoxin, and chemokines, including chemokine (C-X-C motif) ligand (CXCL)8, CXCL1, and chemokine (C-C motif) ligand (CCL) 2 [88, 89]. TSLP increases the expression of intercellular adhesion molecule (ICAM)-1 and CD18, but suppresses the expression of surface L-selectin, suggesting that it plays a role in promoting eosinophil transmigration and accumulation in tissues [89]. The effects of TSLP on eosinophils are mediated through the extracellular signal-regulated kinase (ERK), p38 mitogen-activated protein kinase (MAPK), and nuclear factor κ light chain enhancer of activated B cells (NF-κB) signaling pathways [88, 89]. In addition, TSLP can induce the formation of eosinophil extracellular traps composed of mitochondrial deoxyribonucleic acid (DNA) in association with eosinophil cationic proteins, which play an important role in innate immune responses to infectious agents that subsequently lead to tissue damage in asthmatic airways [90]. Here, TSLP promotes eosinophilia in the airways. Anti-TSLP therapy can significantly reduce the number of eosinophils in blood and sputum in asthma in association with a reduction in bronchial obstruction following allergen exposure [75]. 

This is supported by correlation studies in patients with atopic asthma, in which the level of immunopositive staining for TSLP in bronchial biopsies correlated with airway eosinophilia 24 hours after allergen exposure [51]. In contrast, the concentration of TSLP in the induced sputum of asthma patients during virus-induced exacerbations was inversely related to the number of eosinophils, suggesting different mechanisms of action of TSLP in acute exacerbations compared with chronic eosinophilic inflammation [64]. 

### TSLP and hematopoietic progenitor cells (eosinophil progenitor cells) 

There is evidence of a link between allergic respiratory reactions and the mobilization of bone marrow-derived eosinophil progenitor cells (EoPs). Affected tissues support local differentiation, proliferation, maturation, and activation of EoPs that migrate to the site of allergen exposure in the mucosa during allergic airway disease. TSLP has been shown to promote activation, migration, and local differentiation of EoPs in the airways. Cord blood-derived hematopoietic progenitor cells cultured with TSLP at nanomolar concentrations upregulate IL-5Rα expression and then, in combination with IL-3 or granulocyte-macrophage colony-stimulating factor (GM-CSF), stimulate significant growth of eosinophil/basophil colony-forming units (Eo/Bo-CFUs) [91]. 

In addition, increased eosinophil activity was detected in bronchial epithelial supernatants from patients with severe eosinophilic asthma compared with patients with mild asthma and healthy controls. This activity was attenuated by a receptor-blocking antibody to TSLP [92]. In picogram amounts, TSLP additively stimulated the growth of Eo/Bo CFUs through IL-5 [92]. At the messenger ribonucleic acid (mRNA) level, a synergistic increase in nuclear transcription factor GATA-2 and CCAAT/enhancer-binding protein (CEBP)-α was observed in CD34+ cells in the presence of TSLP and IL-5 [92]. Collectively, these results suggest that eosinophilopoiesis is not driven by IL-5 alone, but rather is a complex process involving the interaction between local and systemically produced growth factors, including TSLP. 

Migration of progenitor cells into the airways is an important component in triggering local eosinophilic inflammation. Prior exposure to TSLP and IL-33 stimulates progenitor cell migration toward the chemoattractant stromal cell-derived factor (SDF)-1α (CXCL12) [93]. This implies that airway epithelium can locally release alarmin cytokines that increase the migratory propensity of CD34+ progenitor cells. Moreover, CD34+ primitive progenitor cells express TSLPR, and stimulation with TSLP results in a dose-dependent release of IL-5, IL-13, GM-CSF, and chemokines such as CCL22, CXCL8, and CCL1 [93, 94]. This suggests that TSLP not only drives local maturation of eosinophil lineage progenitor cells but can also promote proinflammatory function and migration of primitive progenitor cells. 

### TSLP and ILC2s 

ILC2s produce substantial amounts of T2 cytokines such as IL-5, IL-13, and IL-9 after activation by alarmin cytokines such as TSLP, IL-25, and IL-33 [95, 96, 97]. This effect is enhanced by the presence of IL-2 and IL-7 [98]. TSLP, in synergy with IL-25 or IL-33, can promote the production of IL-5 and IL-13 by ILC2s [95] and prolong the survival of ILC2s [98]. Activation of ILC2s by IL-33 and TSLP leads to upregulation of surface receptor expression of tyrosine kinase c-KIT and downregulation of IL-7Rα and chemoattractant receptor homologous molecule expressed on Th2 cells (CRTH2), suggesting that alarmin cytokines can generate heterogeneous populations of ILC2s [98]. The functions of the different populations remain to be elucidated. 

For inflammatory airway diseases, Chen et al. [99] reported that mild asthmatics had a rapid and significant increase in sputum ILC2s within 24 hours of allergen inhalation, expressing large amounts of IL-5 and IL-13. Phenotypic analysis of ILC2s in this study showed upregulation of TSLPR on IL-33 receptor-expressing ILC2s, suggesting that increased responsiveness of ILC2s to TSLP in the airways may contribute to the spread of eosinophilic inflammation. Other studies have shown that the number of ILC2s is increased in patients with severe asthma and persistent eosinophilia compared to patients with mild asthma, with the greatest number of IL-5+IL-13+ILC2s in the airways observed in patients with uncontrolled disease and high eosinophilia despite treatment with high-dose oral corticosteroids [100, 101, 102]. In endobronchial biopsies from prednisone-treated patients with severe asthma, ILC2s were colocalized in TSLP-immunopositive regions [56]. Similarly, the number of ILC2s in nasal biopsies was shown to positively correlate with TSLP levels in nasal tissues of patients with severe asthma and chronic rhinosinusitis [55]. Liu et al. [103] reported that dexamethasone treatment resulted in inhibition of IL-5 production by ILC2s after in vitro stimulation of peripheral blood cultures from patients with severe asthma with *Aspergillus* or IL-2/IL-33. In contrast, dexamethasone had no effect on airway ILC2s, indicating compartmental differences in steroid resistance of ILC2s [103]. This was attributed to higher TSLP levels in the airways. Specifically, the study showed that the inhibitory effect of dexamethasone on ILC2s in the airways was reduced in the presence of TSLP and IL-7. Furthermore, this was found to be dependent on mitogen-activated protein kinase kinases (MEK) and signal transduction and activator of transcription factor 5 (STAT5) signaling [103]. Three genes, *CBX7, MEK2,* and *TRL2,* have been identified in TSLP-stimulated lymphoid cells resistant to dexamethasone treatment [104]. TSLP itself can induce the expression of MEK2, which translocates to the nucleus and interacts with chromobox protein homolog 7 (CBX7), suggesting a positive feedback regulatory pathway [103]. Thus, dexamethasone appears to attenuate the proinflammatory activity of ILC2s triggered by IL-33, and TSLP appears to induce steroid resistance in ILC2s. 

### TSLP and mast cells 

Mast cells play an important role in triggering eosinophilic airway inflammation through IgE-FcεR1 cross-linking, leading to the release of histamine, leukotrienes, and many other cytokines/chemokines. Alarmin cytokines may affect mast cell function. Mast cells express TSLPR and, upon stimulation with TSLP alone or together with IL-1β and TNF-α, produce T2 cytokines and chemokines CXCL8 and CCL1, with no effect on mast cell proliferation or survival [46, 54, 105]. Interestingly, mast cells can produce substantial amounts of TSLP themselves following IgE cross-linking or priming with IL-4 [106, 107]; and crosstalk between smooth muscle cells of the respiratory tract (ASMCs) and mast cells has been reported, as evidenced by chronically activated mast cells triggering the release of TSLP via a TNF-α-dependent pathway. In turn, TSLP derived from ASMCs induced T2 cytokine production by mast cells [108]. Taken together, these studies demonstrate that TSLP can directly interact with mast cells to promote eosinophilic inflammation through the production of T2 cytokines. 

### TSLP and monocytes/macrophages 

Macrophages are leukocytes found throughout the respiratory tract. There are few studies on the effect of TSLP on airway macrophages in humans. Herein, TSLP was shown to increase the expression of the activation marker CD80 in CD14+ monocytes/macrophages, suggesting a role in promoting differentiation into mature macrophages [109]. In addition, cDNA from human monocytes cultured with TSLP and IL-7 showed upregulation of CCL17, CCL18, and CCL22, suggesting that TSLP is a promoter for migration of these effector cells into the airways [110]. In bronchial biopsy tissues, TSLP expression has been shown to colocalize with epithelial CD68+ macrophages in the tissue, with a greater number of macrophages detected in asthma patients compared with control subjects or healthy individuals [56, 57, 58]. 

### TSLP and basophils 

Basophils play an important role in chronic inflammatory airway disease as a significant source of T2 cytokines, including IL-4, IL-13, and pro-inflammatory mediators such as histamine and leukotrienes. Basophil development, homeostasis, and function are largely regulated by IL-3, but there is increasing evidence that TSLP also affects basophil differentiation. 

Peripheral blood-derived CD34+ cells incubated with IL-3 and TNF-α prior to incubation have increased sensitivity to TSLP-mediated basophil differentiation [91]. In addition, bone marrow mesenchymal stromal cells produce TSLP. These are activated by mast cells and promote the differentiation of CD34+ progenitor cells into Eo/Bo CFUs [111]. Mature basophils express TSLPR, which is upregulated in the presence of IL-3 [112]. In comparison, TSLP-stimulated basophils show greater expression of the IL-33 receptor ST2, indicating the existence of heterogeneous basophil populations [112]. Allergen stimulation of peripheral blood mononuclear cells in patients with atopic dermatitis resulted in upregulation of TSLPR on basophils and myeloid dendritic cells, which was further enhanced by IgE-FcεR1 cross-linking [113]. 

In blood samples from patients with allergic asthma, significant upregulation of TSLPR on basophils was found after direct stimulation with anti-IgE antibodies, which correlated with total serum IgE [114]. However, another study in asthma patients reported that stimulation with anti-IgE increased the expression of IL-25 and IL-33 receptors, but not TSLPR [115]. These studies suggest that there may be both IgE-dependent and IgE-independent mechanisms that increase basophil responsiveness to TSLP. Basophil TSLPR expression is significantly increased after respiratory allergen exposure [116]. In addition, TSLP stimulation of peripheral basophils increased activation marker (CD203 c) expression, T2 cytokine production, histamine release, and eotaxin-induced cellular migration responses [116]. Stimulation of basophils with TSLP also increases IL-25 receptor (IL-17RB) and ST2 expression, suggesting that TSLP may increase basophil response to other alarmin cytokines [117]. 

Thus, TSLP is an important mediator of the basophil inflammatory response. 

### TSLP and dendritic cells 

Human myeloid dendritic cells express TSLPR [118], and stimulation with TSLP can directly upregulate the expression of major histocompatibility complex class II and co-stimulatory molecules CD40, CD86, CD54, CD90, CD83, and dendritic cell lysosome-associated membrane protein (DC-LAMP), as well as chemokines CXCL8, CCL24, CCL17, CCL22, and CCL1 [48, 119, 120, 121]. Interestingly, monocyte-derived dendritic cells can themselves produce TSLP upon stimulation by microbial products, suggesting that TSLP may act in an autocrine manner to further drive T2 inflammation [122, 123]. TSLP is also an important driver of dendritic cell-mediated T-cell differentiation [124]. Even in the absence of IL-12, TSLP can induce the expression of OX40 ligand (OX40 L) [120]. OX40 L is expressed by TSLP-induced dendritic cells and leads to the differentiation of naive CD4+ T cells into TNF-α+IL-10 T helper cells (Th2 cells) [120]. OX40 L can convert IL-10-producing regulatory Th1 cells, into TNF-α-producing Th2 cells, thus OX40 L can act as a Th2-polarizing signal [120, 125]. A combination of TSLP and allergen stimulates peripheral myeloid dendritic cells of patients with inhalant allergy to induce differentiation of CD4+ T cells into Th2 cells, whereas TSLP alone promotes polarization into Th9 cells [126]. Expression of OX40 L is required for induction of Th2 polarization but not for Th9 polarization (Th9 cells require the presence of transforming growth factor (TGF)-β1 [126]. Exosomes produced by TSLP-activated dendritic cells express OX40 L, which promotes CD4+ T-cell proliferation and IL-4 production [127]. TSLP has a priming effect on myeloid dendritic cell-mediated expansion and function of CRTH2+ and CD4+ memory Th2 cells and inhibits the development of forkhead box protein-3 (FOXP3) positive regulatory T cells (Treg) [128, 129]. These studies demonstrate that the interaction between dendritic cells and TSLP is an important triggering event leading to the promotion of naïve T-cell differentiation and polarization and downstream T2 inflammation, which is mediated in part by OX40 L. 

### TSLP and lymphocytes 

Most studies on the influence of TSLP on lymphocytes referred to the indirect effect of TSLP through dendritic cells on T-cell differentiation [120, 124, 126]; however, evidence exists that TSLP can directly modulate human T lymphocytes. In resting CD4+ T cells, TSLPR is minimally expressed; however, upon their activation, TSLPR expression increases significantly [130]. TSLP can promote proliferation and differentiation of naive CD4+ T cells into Th2 cells or memory T cells in the presence of T-cell receptor (TCR) stimulation or IL-4 [130, 131]. Similar effects are observed in CD8+ T cells, whose proliferation TSLP can activate through TCR stimulation [132]. 

The direct effects of TSLP on Tregs have not been well studied. Tregs express TSLPR, and stimulation with TSLP impairs IL-10 production [128]. Immunosuppressive Treg activity is reduced in patients with allergic airway disease in both adults and children [60, 128]. These findings suggest that TSLP may reduce the anti-inflammatory function of Tregs and thus further increase T2 inflammation. Overall, these data suggest that TSLP can directly modulate T lymphocytes, leading to downstream T2 inflammation and airway eosinophilia. 

## Mechanisms of action of TSLP in non-T2 inflammation 

Non-T2 inflammation in CRS is often mediated by Th17 cells and neutrophils. IL-17A produced by Th17 cells stimulates airway epithelial cells to produce neutrophilia-promoting cytokines such as CXCL8 (IL-8) and GM-CSF [133] and promotes airway remodeling by altering the function of ASMCs [134]. 

TSLP enhances Toll-like receptor (TLR) 3 ligand-induced production of IL-23 by dendritic cells and induces programming of naïve CD4+ T cells into Th17 cells [135]. In addition, TSLP stimulates dendritic cells to Th2 and Th17 polarization, as evidenced by an increase in IL-4+/IL-17A+ T cells and upregulation of IL-4/IL-17A protein levels such as IL-6 and IL-23 in cell culture supernatant [136]. These results suggest that TSLP and TLR3 ligands promote Th17 cell differentiation under Th2-polarizing conditions by activating dendritic cells. TSLP can also activate neutrophils by directly interfering with the C5 complement system and modulating neutrophil production of reactive oxygen species [137]. 

In chronic inflammatory airway disease, levels of IL-33 and TSLP, but not IL-25, were significantly elevated compared to control subjects [63]. Previous studies had shown that neutrophils are a source of TSLP in bronchial biopsy tissue [57, 58, 138]. In this light, it seems interesting that anti-TSLP monoclonal antibody therapy reduced exacerbations in patients with severe asthma with non-T2 inflammation, and thus TSLP may play a role in patients with little or no T2 inflammation [139]. The prevalence of TSLP in other respiratory diseases, such as chronic obstructive pulmonary disease [57], also suggests that TSLP may be involved in other T2-independent inflammatory pathways. 

## Epithelial mechanisms of action of TSLP 

In addition to its effects on specific immune cells, there is now ample evidence that TSLP serves as a mediator between immune cells and epithelial cells in the airways. Dysregulation of the epithelium leads to characteristic airway changes known as airway remodeling, which include reticular basement membrane thickening, goblet cell hyperplasia, subepithelial fibrosis, and ASMC hyperplasia and/or hypertrophy [140]. 

### TSLP and ASMCs 

Numerous reports indicate that TSLP is an important modulator of ASMC activity. Human ASMCs express TSLPR [54], and stimulation with TSLP leads to the expression of IL-6, CCL11, and CXCL8, as well as migration through STAT3 signaling [141, 142, 143, 144]. ASMCs are a significant source of TSLP [54, 144, 145], which is enhanced in the presence of TNF-α and IL-1β via the p38 and MAPK signaling pathways [142, 146]. TNF-α and IL-1β can promote TSLP expression in ASMCs in healthy individuals, and activated mast cells can induce TSLP release in ASMCs [108]. These results suggest that TSLP can promote airway inflammation through an interaction between mast cells and airway epithelial cells [46, 57, 108]. 

### TSLP and fibroblasts 

TSLP can promote airway remodeling in chronic inflammatory airway diseases through activation of fibroblasts [147] in terms of significant production of collagen and α-smooth muscle actin via a p38 MAPK- and STAT3-dependent pathway [148, 149]. Furthermore, TSLP expression in bronchial biopsy tissue has been shown to be restricted to fibroblasts [138, 145, 150]. Specifically, TSLP has been shown to increase the production of TGF-β1 and arginase 1 by fibroblasts at the mRNA and protein levels [151]. 

## What is the benefit of TSLP blockade in patients with chronic respiratory disease? Clinical trial data 

The TSLPR complex has been associated with a number of chronic *inflammatory* airway diseases [152]. Two monoclonal antibodies designed to block TSLP and thus inhibit signaling through TSLPR are being investigated in clinical trials as drugs for the treatment of asthma. Tezepelumab was initially tested as an intravenous formulation and subsequently also tested in the subcutaneous dosage form. 

CSJ117 is a fully human neutralizing Fab fragment (antibody-antigen binding fragment: Fab) belonging to the IgG1/λ isotype subclass. CSJ117 was developed as an inhaled formulation for targeted administration to the lung to bind to TSLP released from airway epithelial cells [44, 45]. 

GSK2618960 is a humanized IgG1 monoclonal antibody directed against the alpha component (IL-7Rα; CD127) of TSLPR [153] and is being developed for the treatment of autoimmune diseases (including multiple sclerosis) [154]. 

In patients with severe eosinophilic airway inflammation [155], blocking IL-7Rα may be helpful. Intravenous administration of GSK2618960 resulted in more than 95% receptor occupancy on CD3+ T cells and effectively blocked IL-7 receptor signaling as measured by STAT5 phosphorylation [154]. 

### Tezepelumab treatment resulted in positive outcomes in asthma in two clinical trials 

In a double-blind placebo-controlled phase 1b study to demonstrate the efficacy of tezepelumab in an allergen provocation chamber [75], tezepelumab was administered intravenously at a dose of 700 mg every 4 weeks for 3 months in adults with allergic asthma. In the actively treated group, blood eosinophil counts began to decrease after 2 weeks and reached normal values after 4 weeks. Sputum eosinophils showed significant improvement into the normal range (< 2%) by the first measurement time point 6 weeks after the first dose. Remarkably, fractional exhaled nitric oxide (FeNO) values improved significantly as early as 1 week after the first dose. On days 42 and 84, inhaled allergen challenge was performed to elicit eosinophilic inflammation in the airways; tezepelumab significantly inhibited allergen-induced early and late asthmatic responses, as well as post-challenge inflammation levels, including FeNO, and blood and sputum eosinophils. Systemic treatment was found to be effective in regulating both circulating and local inflammatory levels. 

The second completed study in asthma (PATHWAY) was a large, multicenter, randomized, parallel-group, double-blind, placebo-controlled phase 2 trial [139]. The study evaluated the efficacy and safety of tezepelumab as an add-on therapy for patients with moderate-to-severe asthma and a history of exacerbations and uncontrolled disease who were receiving inhaled corticosteroids and long-acting β2-agonists with or without oral corticosteroids and additional asthma controllers. Three tezepelumab dosing regimens were studied: low (70 mg every 4 weeks), medium (210 mg every 4 weeks), and high (280 mg every 2 weeks), administered subcutaneously for 1 year. The study found significant reductions in annual exacerbation rates of 62, 71, and 66%, respectively, in the low, medium, and high tezepelumab dose groups compared with placebo, along with significant improvements in lung function and inflammatory markers (blood FeNO and eosinophils) in all active treatment groups. Interestingly, these improvements were observed regardless of patient phenotype and independent of peripheral blood eosinophil counts, IgE levels, and FeNO levels, suggesting that tezepelumab has similar efficacy in patients with T2-related and non-T2-related disease. In addition, proinflammatory biomarkers and the proteome were examined. In the cohort receiving tezepelumab at 210 mg every 4 weeks (the dose chosen for the phase 3 trials), serum IL-5 and IL-13 levels and blood eosinophil counts decreased by at least 50% at 1 year compared to baseline, along with 25 and 20% reductions in FeNO and total IgE, respectively [156]. Proteomic analyses revealed a reduction in proteins associated with matrix remodeling (MMP-10 and periostin), demonstrating a broad biological effect of TSLP blockade [157]. 

In addition to the studies with tezepelumab, a multinational proof-of-concept study of CSJ117 in the allergen challenge model was conducted in patients with mild allergic asthma to evaluate safety, tolerability, pharmacokinetics, and pharmacodynamics. 28 participants completed the study, which included daily inhalation of CSJ117 and bronchial allergen challenges at 6 and 12 weeks. The study was completed in 2019; results are pending [158]. 

Additional clinical trials evaluating the efficacy, mechanisms of action, and long-term safety of tezepelumab are ongoing. Two pivotal phase 3 trials (NAVIGATOR and SOURCE) are being conducted in patients with severe asthma receiving inhaled corticosteroids/long-acting β-agonists with or without oral corticosteroids for maintenance therapy and additional asthma controllers [159, 160]. The primary endpoints were reduction in asthma exacerbation rate or daily oral corticosteroids. Another bronchoscopy study (CASCADE) aims to better understand the mechanisms of TSLP blockade by investigating the effects of tezepelumab on the number of inflammatory cells in endobronchial biopsies from adults with inadequately controlled moderate-to-severe asthma [161]. Data on long-term safety and tolerability will be important and are currently being investigated in a tezepelumab extension study [162]. Taken together, these studies will provide much needed information on the benefits of TSLP blockade in asthma. 

Tezepelumab is also tested in a phase III study for CRSwNP. The WAYPOINT study recently started recruiting patients. However, the first study results are not expected until 2024. 

## Discussion and conclusion 

Two biologics have now been approved in Germany (dupilumab and omalizumab) as an add-on treatment for severe, uncontrolled chronic rhinosinusitis with nasal polyps. Based on positive study data, it is likely that two additional compounds will also receive approval in the future (mepolizumab and benralizumab) [4, 163]. All of these biologics are targeting T2 inflammation. 

TSLP plays a central role in chronic airway mucosal inflammation. TSLP is also mostly involved in T2 inflammation, but quite possibly also in non-T2 inflammation, and has multiple effects on a variety of cell types including ILC2s, hematopoietic progenitor cells, eosinophils, basophils, mast cells, monocytes/macrophages, dendritic cells, lymphocytes, neutrophils, smooth muscle cells, and fibroblasts [5]. 

Treatment options for chronic inflammatory airway diseases have improved significantly in recent years with the introduction of T2-targeting biologics. 

They act on different targets of T2 inflammation, for example IgE, IL-5, IL-4/IL-13. By activating several downstream inflammatory pathways, TSLP affects airway inflammation more comprehensively than the targets of previously approved biologics, which is why anti-TSLP-targeted biologics can sustainably improve epithelial immune regulation in chronic inflammatory airway disease [5]. 

TSLP blockade has shown promise in treating both T2-related and non-T2-related (i.e., non-allergic, non-eosinophilic) inflammation in asthma when administered over a period of up to 1 year. For patients with non-T2-related inflammation, this would be the first therapeutic option with monoclonal antibodies. 

As with other biologics, it will be important to find biomarkers that identify patients with a good chance of success with anti-TSLP therapy. Blood eosinophils, serum IgE, and FeNO have been used as biomarkers to analyze treatment with anti-IL-5/IL-5Rα, anti-IgE, and anti-IL-4/IL-13 monoclonal antibodies. While TSLP itself could hypothetically be used as a biomarker, this has not been shown to be effective, primarily because of the difficulty in measuring low concentrations of this cytokine [5]. 

In addition, TSLP has been shown to be cleaved in nasal polyp tissue by endogenous proteases, resulting in bioactive peptides [164, 165], making anti-TSLP antibodies undetectable in ex vivo studies and possibly underestimating actual TSLP production [5]. 

The clinical relevance of systemic TSLP detection is also unclear, as there are large variations in peripheral blood independent of disease activity in the airways [5]. 

Attempts to quantify TSLP in patient samples are also complicated by the fact that there are two isoforms of the protein: a long-form TSLP (lfTSLP), in which the protein is full-length, and a form that is approximately half amino acid length (63 amino acids), often referred to as short-form TSLP (sfTSLP) [166, 167, 168, 169]. It is currently unknown whether anti-TSLP therapies in clinical development bind to lfTSLP, sfTSLP, or both. While the role of lfTSLP is well characterized, the function of sfTSLP remains unclear. It is thought to be constitutively expressed in human tissues but not in rodents [170, 171]. In addition, it does not bind to the TSLPR complex [170, 172], suggesting a different biological function than lfTSLP. The relative ratio of lfTSLP to sfTSLP has not yet been determined in patients with chronic respiratory disease, in part because no research reagents to distinguish the two forms of TSLP are available. Currently, lfTSLP and sfTSLP can only be distinguished at the mRNA level using specific primers. Such studies examining the two isoforms in human tissues have shown that the long isoform of TSLP is proinflammatory and expressed in inflammation [173], and that the ratio of TSLP isoforms may be altered in different inflammatory diseases [172]. Further research is needed to better understand the role of the two TSLP isoforms, their regulation by SNPs, and their expression under different pathological conditions. 

Although TSLP is primarily expressed by epithelial cells at barrier surfaces (nose, nasal sinuses, lung, intestine, skin), TSLP can also be produced by a variety of immune cells; therefore, systemic administration of anti-TSLP also has the potential to disrupt other homeostatic functions of TSLP [172, 174]. The long-term safety and efficacy of anti-TSLP treatment must therefore be evaluated, ideally considering not only T2-related and non-T2-related inflammation, but also TSLP variants, gene polymorphisms, and ethnically diverse populations. In addition, inhaled TSLP blockade, which directly targets TSLP produced in the airways, is an interesting alternative route that could be important for the safety and tolerability of anti-TSLP therapy. 

This is especially true in situations such as the current COVID-19 pandemic, where continuing or reinitiating biologic therapy for CRSwNP is recommended even during SARS-CoV-2 virus infection or in the setting of a planned COVID-19 vaccination [175]. The timing of vaccination should be chosen to be midway between two biologic injections. Depending on the dosing interval and preparation, this corresponds to 1 – 2 weeks (for dupilumab and omalizumab), with a minimum interval of 1 week recommended [175]. Recommendations have also been developed for potential adverse effects of biologics therapy in the setting of COVID-19 vaccination [176] and for the management of patients at risk of anaphylaxis [177]. 

## Take-home messages

- Because of the common T2 endotype, most of the biologics developed for asthma are also suitable for CRSwNP.
- TSLP expression in asthma patients correlates with both airway obstruction and the severity of the disease.
- TSLP promotes eosinophilia in the airways.
- The migration of progenitor cells into the airway is an important component in the process triggering local eosinophilic inflammation.
- TSLP interacts directly with mast cells to promote eosinophilic inflammation through the production of T2 cytokines.
- TSLP is also a key mediator of the basophilic inflammatory response.
- There is also evidence that TSLP can directly modulate human T lymphocytes.
- The expression of TSLP in bronchial biopsy tissue is limited to fibroblasts.
- There is evidence that tezepelumab has similar effectiveness in patients with T2-related and those with non-T2 disease.
- TSLP is involved in both T2 inflammation and non-T2 inflammation and shows effects on a wide variety of cell types.

## Funding 

None.

## Conflict of interest 

L. Klimek reports grants and/or honoraria from Allergopharma, MEDA/Mylan, HAL Allergie, ALK Abelló, LETI Pharma, Stallergenes, Quintiles, Sanofi, ASIT Biotech, Lofarma, Allergy Therapeut., AstraZeneca, GSK, Inmunotk, outside the submitted work; and memberships in the following organizations: AeDA, DGHNO, German Academy of Allergology and Clinical Immunology, ENT-BV GPA, EAACI. 

J. Hagemann states to have received grants from Novartis Pharma GmbH for lectures. 

U. Förster-Ruhrmann received honoraria for lectures from Novartis, AstraZeneca, Sanofi, and GSK, outside the present work. 

Ch. Bergmann, P.V. Tomazic, C. A. Hintschich, T. Huppertz, H.J. Welkoborsky, M. Cuevas, I. Casper, S. Becker, have no conflicts of interest related to this work. 

## References

[1] Akdis CA Does the epithelial barrier hypothesis explain the increase in allergy, autoimmunity and other chronic conditions? Nat Rev Immunol. 2021; 21: 739–751. 33846604 10.1038/s41577-021-00538-7

[2] Klimek L Casper I Siemer S Wollenberg B Stauber R Koennecke M [T-cell immune responses in chronic inflammatory diseases of the nasal mucosa]. HNO. 2019; 67: 881–892. 31598772 10.1007/s00106-019-00759-2

[3] Klimek L Casper I Wollenberg B Stauber R Koennecke M [Histamine receptors in chronic inflammatory diseases of the nose and paranasal sinuses]. HNO. 2019; 67: 389–400. 30944947 10.1007/s00106-019-0649-z

[4] Koennecke M Klimek L Mullol J Gevaert P Wollenberg B Subtyping of polyposis nasi: phenotypes, endotypes and comorbidities. Allergo J Int. 2018; 27: 56–65. 29564208 10.1007/s40629-017-0048-5 PMC5842507

[5] Gauvreau GM Sehmi R Ambrose CS Griffiths JM Thymic stromal lymphopoietin: its role and potential as a therapeutic target in asthma. Expert Opin Ther Targets. 2020; 24: 777–792. 32567399 10.1080/14728222.2020.1783242

[6] Hastan D Fokkens WJ Bachert C Newson RB Bislimovska J Bockelbrink A Bousquet PJ Brozek G Bruno A Dahlén SE Forsberg B Gunnbjörnsdóttir M Kasper L Krämer U Kowalski ML Lange B Lundbäck B Salagean E Todo-Bom A Tomassen P Chronic rhinosinusitis in Europe – an underestimated disease. A GA LEN study. Allergy. 2011; 66: 1216–1223. 21605125 10.1111/j.1398-9995.2011.02646.x

[7] Hirsch AG Stewart WF Sundaresan AS Young AJ Kennedy TL Scott Greene J Feng W Tan BK Schleimer RP Kern RC Lidder A Schwartz BS Nasal and sinus symptoms and chronic rhinosinusitis in a population-based sample. Allergy. 2017; 72: 274–281. 27590749 10.1111/all.13042 PMC5497579

[8] Ostovar A Fokkens WJ Vahdat K Raeisi A Mallahzadeh A Farrokhi S Epidemiology of chronic rhinosinusitis in Bushehr, southwestern region of Iran: a GA2LEN study. Rhinology. 2019; 57: 43–48. 30033451 10.4193/Rhin18.061

[9] Stuck BA Beule A Jobst D Klimek L Laudien M Lell M Vogl TJ Popert U [Guideline for “rhinosinusitis”-long version : S2k guideline of the German College of General Practitioners and Family Physicians and the German Society for Oto-Rhino-Laryngology, Head and Neck Surgery]. HNO. 2018; 66: 38–74. 28861645 10.1007/s00106-017-0401-5

[10] Fokkens WJ Lund VJ Mullol J European Position Paper on rhinosinusitis and nasal polyps 2012. Rhinol Suppl. 2012; 23: 3 p preceding table of contents, 1–298. 22764607

[11] Fokkens WJ Lund VJ Mullol J Bachert C Alobid I Baroody F Cohen N Cervin A Douglas R Gevaert P Georgalas C Goossens H Harvey R Hellings P Hopkins C Jones N Joos G Kalogjera L Kern B Kowalski M EPOS 2012: European position paper on rhinosinusitis and nasal polyps 2012. A summary for otorhinolaryngologists. Rhinology. 2012; 50: 1–12. 22469599 10.4193/Rhino12.000

[12] Rosenfeld RM Clinical practice guideline on adult sinusitis. Otolaryngol Head Neck Surg. 2007; 137: 365–377. 17765760 10.1016/j.otohns.2007.07.021

[13] Agarwal A Spath D Sherris DA Kita H Ponikau JU Therapeutic antibodies for nasal polyposis treatment: Where are we headed? Clin Rev Allergy Immunol. 2020; 59: 141–149. 31073812 10.1007/s12016-019-08734-z

[14] Franzese CB The role of biologics in the treatment of nasal polyps. Immunol Allergy Clin North Am. 2020; 40: 295–302. 32278452 10.1016/j.iac.2019.12.006

[15] Klimek L Förster-Ruhrmann U Becker S Chaker A Strieth S Hoffmann TK Dazert S Deitmer T Olze H Glien A Plontke S Wrede H Schlenter W Welkoborsky HJ Wollenberg B Beule AG Rudack C Wagenmann M Stöver T Huppertz T Positionspapier: Anwendung von Biologika bei chronischer Rhinosinusitis mit Polyposis nasi (CRSwNP) im deutschen Gesundheitssystem – Empfehlungen des Ärzteverbandes Deutscher Allergologen (AeDA) und der AGs Klinische Immunologie, Allergologie und Umweltmedizin und Rhinologie und Rhinochirurgie der Deutschen Gesellschaft für HNO-Heilkunde, Kopf- und Halschirurgie (DGHNOKHC). Laryngorhinootologie. 2020; 99: 511–527. 32575138 10.1055/a-1197-0136

[16] Klimek L Koennecke M Hagemann J Wollenberg B Becker S [Immunology of chronic rhinosinusitis with nasal polyps as a basis for treatment with biologicals]. HNO. 2019; 67: 15–26. 30167718 10.1007/s00106-018-0557-7

[17] Jutel M Van de Veen W Agache I Azkur KA Akdis M Akdis CA Mechanisms of allergen-specific immunotherapy and novel ways for vaccine development. Allergol Int. 2013; 62: 425–433. 24153333 10.2332/allergolint.13-RAI-0608

[18] Klimek L Bachert C Pfaar O Becker S Bieber T Brehler R Buhl R Casper I Chaker A Czech W Fischer J Fuchs T Gerstlauer M Hörmann K Jakob T Jung K Kopp MV Mahler V Merk H Mülleneisen N ARIA guideline 2019: treatment of allergic rhinitis in the German health system. Allergol Select. 2019; 3: 22–50. 32176226 10.5414/ALX02120E PMC7066682

[19] Klimek L Brehler R Hamelmann E et al. Development of subcutaneous allergen immunotherapy (part 2): preventive aspects and innovations / CME-Fragebogen. Allergo-Journal. 2019;28*:* 31-45.

[20] Klimek L Brehler R Hamelmann E et al. Evolution of subcutaneous allergen immunotherapy (part 1): from first developments to mechanism-driven therapy concepts. Allergo J Int 2019; 28 *:* 78-95.

[21] Klimek L Casper I Bergmann KC Biedermann T Bousquet J Hellings P Jung K Merk H Olze H Mösges R Schlenter W Gröger M Ring J Chaker A Pfaar O Wehrmann W Zuberbier T Becker S Positionspapier. Die Therapie der allergischen Rhinitis in der Routineversorgung: evidenzbasierte Nutzenbewertung der kombinierten Anwendung mehrerer Wirkstoffe. Allergologie. 2020; 43: 476–488.

[22] Ade R Rehm M A prediction of dust mite populations in different categories of housing quality in Auckland, New Zealand. Allergo J Int. 2020; 29: 187–198.

[23] Pitsios C Pantavou K Terreehorst I Cianferoni A Nowak-Wegzryn A Vidal C Vassilopoulou E Papachristodoulou M Tsigkrelis GP Bonovas S Nikolopoulos GK Use of allergy tests to identify dietary and environmental triggers of eosinophilic esophagitis: protocol for a systematic review. Allergo J Int. 2020; 29: 280–283.

[24] McDowell PJ Heaney LG Different endotypes and phenotypes drive the heterogeneity in severe asthma. Allergy. 2020; 75: 302–310. 31267562 10.1111/all.13966

[25] Tran TN Zeiger RS Peters SP Overlap of atopic, eosinophilic, and TH2-high asthma phenotypes in a general population with current asthma. Annals of allergy, asthma & immunology: official publication of the American College of Allergy, Asthma, &. Immunology. 2016; 116: 37–42. 10.1016/j.anai.2015.10.027 26707771

[26] Wenzel SE Asthma phenotypes: the evolution from clinical to molecular approaches. Nat Med. 2012; 18: 716–725. 22561835 10.1038/nm.2678

[27] Woodruff PG Modrek B Choy DF Jia G Abbas AR Ellwanger A Koth LL Arron JR Fahy JV T-helper type 2-driven inflammation defines major subphenotypes of asthma. Am J Respir Crit Care Med. 2009; 180: 388–395. 19483109 10.1164/rccm.200903-0392OC PMC2742757

[28] Agache I Song Y Alonso-Coello P Vogel Y Rocha C Solà I Santero M Akdis CA Akdis M Canonica GW Chivato T Del Giacco S Eiwegger T Fokkens W Georgalas C Gevaert P Hopkins C Klimek L Lund V Naclerio R Efficacy and safety of treatment with biologicals for severe chronic rhinosinusitis with nasal polyps: A systematic review for the EAACI guidelines. Allergy. 2021; 76: 2337–2353. 33683704 10.1111/all.14809

[29] Fokkens WJ Lund V Bachert C Mullol J Bjermer L Bousquet J Canonica GW Deneyer L Desrosiers M Diamant Z Han J Heffler E Hopkins C Jankowski R Joos G Knill A Lee J Lee SE Mariën G Pugin B EUFOREA consensus on biologics for CRSwNP with or without asthma. Allergy. 2019; 74: 2312–2319. 31090937 10.1111/all.13875 PMC6972984

[30] Gülsen A Wedi B Jappe U Hypersensitivity reactions to biologics (part I): allergy as an important differential diagnosis in complex immune-derived adverse events. Allergo J Int. 2020; 29: 97–125. 32421085 10.1007/s40629-020-00126-6 PMC7223134

[31] Gülsen A Wedi B Jappe U Hypersensitivity reactions to biologics (part II): classifications and current diagnostic and treatment approaches. Allergo J Int. 2020; 29: 139–154. 10.1007/s40629-020-00126-6 PMC7223134 32421085

[32] Romano C Pace G Sellitto A Reginelli A Omalizumab as a last resort therapy for intractable, severe chronic allergic rhinosinusi-tis. Allergo J Int. 2019; 28: 99–102.

[33] Klimek L Pfaar O Worm M Anwendung von Biologika bei allergischen und Typ-2-entzündlichen Erkrankungen in der aktuel-len COVID-19-Pandemie – ein Positionspapier von AeDA DGAKI GPA ÖGAI LGAI ÖGP ARIA und EAACI. Use of biologicals in allergic and type-2 inflammatory diseases in times of the current COVID-19 pandemic – Position paper of AeDA, DGAKI, GPA, ÖGAI, LGAI, ÖGP, ARIA und EAACI. Allergologie. 2020; 43: 255–271.

[34] Carr TF Zeki AA Kraft M Eosinophilic and Noneosinophilic Asthma. Am J Respir Crit Care Med. 2018; 197: 22–37. 28910134 10.1164/rccm.201611-2232PP PMC5765385

[35] Moore WC Hastie AT Li X Li H Busse WW Jarjour NN Wenzel SE Peters SP Meyers DA Bleecker ER Sputum neutrophil counts are associated with more severe asthma phenotypes using cluster analysis. J Allergy Clin Immunol. 2014; 133: 1557–63.e5. 24332216 10.1016/j.jaci.2013.10.011 PMC4040309

[36] Tliba O Panettieri RA Paucigranulocytic asthma: Uncoupling of airway obstruction from inflammation. J Allergy Clin Immunol. 2019; 143: 1287–1294. 29928921 10.1016/j.jaci.2018.06.008 PMC6301131

[37] Chung KF Wenzel SE Brozek JL Bush A Castro M Sterk PJ Adcock IM Bateman ED Bel EH Bleecker ER Boulet LP Brightling C Chanez P Dahlen SE Djukanovic R Frey U Gaga M Gibson P Hamid Q Jajour NN International ERS/ATS guidelines on definition, evaluation and treatment of severe asthma. Eur Respir J. 2014; 43: 343–373. 24337046 10.1183/09031936.00202013

[38] GINA Global Strategy for Asthma Management and Prevention (2019 update).. In: www.ginasthma.org 2019.

[39] Castro M Corren J Pavord ID Maspero J Wenzel S Rabe KF Busse WW Ford L Sher L FitzGerald JM Katelaris C Tohda Y Zhang B Staudinger H Pirozzi G Amin N Ruddy M Akinlade B Khan A Chao J Dupilumab efficacy and safety in moder-ate-to-severe uncontrolled asthma. N Engl J Med. 2018; 378: 2486–2496. 29782217 10.1056/NEJMoa1804092

[40] Farne HA Wilson A Powell C Bax L Milan SJ Anti-IL5 therapies for asthma. Cochrane Database Syst Rev. 2017; 9: CD010834. 28933516 10.1002/14651858.CD010834.pub3 PMC6483800

[41] Hanania NA Wenzel S Rosén K Hsieh HJ Mosesova S Choy DF Lal P Arron JR Harris JM Busse W Exploring the effects of omalizumab in allergic asthma: an analysis of biomarkers in the EXTRA study. Am J Respir Crit Care Med. 2013; 187: 804–811. 23471469 10.1164/rccm.201208-1414OC

[42] Normansell R Walker S Milan SJ Walters EH Nair P Omalizumab for asthma in adults and children. Cochrane Database Syst Rev. 2014; CD003559. 24414989 10.1002/14651858.CD003559.pub4 PMC10981784

[43] Zayed Y Kheiri B Banifadel M Hicks M Aburahma A Hamid K Bachuwa G Chandran A Dupilumab safety and efficacy in uncontrolled asthma: a systematic review and meta-analysis of randomized clinical trials. J Asthma. 2019; 56: 1110–1119. 30273510 10.1080/02770903.2018.1520865

[44] Corren J Ziegler SF TSLP: from allergy to cancer. Nat Immunol. 2019; 20: 1603–1609. 31745338 10.1038/s41590-019-0524-9

[45] Mitchell PD O’Byrne PM Epithelial-derived cytokines in asthma. Chest. 2017; 151: 1338–1344. 27818325 10.1016/j.chest.2016.10.042

[46] Allakhverdi Z Comeau MR Jessup HK Yoon BR Brewer A Chartier S Paquette N Ziegler SF Sarfati M Delespesse G Thymic stromal lymphopoietin is released by human epithelial cells in response to microbes, trauma, or inflammation and potently activates mast cells. J Exp Med. 2007; 204: 253–258. 17242164 10.1084/jem.20062211 PMC2118732

[47] Kitajima M Lee H-C Nakayama T Ziegler SF TSLP enhances the function of helper type 2 cells. Eur J Immunol. 2011; 41: 1862–1871. 21484783 10.1002/eji.201041195 PMC3124605

[48] Soumelis V Reche PA Kanzler H Yuan W Edward G Homey B Gilliet M Ho S Antonenko S Lauerma A Smith K Gorman D Zurawski S Abrams J Menon S McClanahan T de Waal-Malefyt Rd R Bazan F Kastelein RA Liu YJ Human epithelial cells trigger dendritic cell mediated allergic inflammation by producing TSLP. Nat Immunol. 2002; 3: 673–680. 12055625 10.1038/ni805

[49] Zhang Y Zhou B Functions of thymic stromal lymphopoietin in immunity and disease. Immunol Res. 2012; 52: 211–223. 22274860 10.1007/s12026-012-8264-z PMC3350568

[50] Ziegler SF Roan F Bell BD Stoklasek TA Kitajima M Han H The biology of thymic stromal lymphopoietin (TSLP). Adv Pharmacol. 2013; 66: 129–155. 23433457 10.1016/B978-0-12-404717-4.00004-4 PMC4169878

[51] Al-Sajee D Sehmi R Hawke TJ El-Gammal A Howie KJ Watson RM Londei M Gauvreau GM O’Byrne PM Expression of IL-33 and TSLP and their receptors in asthmatic airways after inhaled allergen challenge. Am J Respir Crit Care Med. 2018; 198: 805–807. 29733677 10.1164/rccm.201712-2468LE

[52] Bleck B Kazeros A Bakal K Garcia-Medina L Adams A Liu M Lee RA Tse DB Chiu A Grunig G Egan JP Reibman J Coexpression of type 2 immune targets in sputum-derived epithelial and dendritic cells from asthmatic subjects. J Allergy Clin Immunol. 2015; 136: 619–627.e5. 25813919 10.1016/j.jaci.2014.12.1950

[53] Ferreira DS Annoni R Silva LFF Buttignol M Santos AB Medeiros MC Andrade LN Yick CY Sterk PJ Sampaio JL Dolhnikoff M Wenzel SE Mauad T Toll-like receptors 2, 3 and 4 and thymic stromal lymphopoietin expression in fatal asthma. Clin Exp Allergy. 2012; 42: 1459–1471. 22994343 10.1111/j.1365-2222.2012.04047.x PMC3459227

[54] Kaur D Doe C Woodman L Heidi Wan WY Sutcliffe A Hollins F Brightling C Mast cell-airway smooth muscle crosstalk: the role of thymic stromal lymphopoietin. Chest. 2012; 142: 76–85. 22052771 10.1378/chest.11-1782 PMC3418864

[55] Lee T-J Fu C-H Wang C-H Huang CC Huang CC Chang PH Chen YW Wu CC Wu CL Kuo HP Impact of chronic rhinosinusitis on severe asthma patients. PLoS One. 2017; 12: 104-111.e101-109 10.1371/journal.pone.0171047 PMC5310870 28199345

[56] Shikotra A Choy DF Ohri CM Increased expression of immunoreactive thymic stromal lymphopoietin in patients with severe asthma. J Allergy Clin Immunol. 2012; 129: 104-111.e101–109. 21975173 10.1016/j.jaci.2011.08.031

[57] Ying S O’Connor B Ratoff J Meng Q Fang C Cousins D Zhang G Gu S Gao Z Shamji B Edwards MJ Lee TH Corrigan CJ Expression and cellular provenance of thymic stromal lymphopoietin and chemokines in patients with severe asthma and chronic obstructive pulmonary disease. J Immunol. 2008; 181: 2790–2798. 18684970 10.4049/jimmunol.181.4.2790

[58] Ying S O’Connor B Ratoff J Meng Q Mallett K Cousins D Robinson D Zhang G Zhao J Lee TH Corrigan C Thymic stromal lymphopoietin expression is increased in asthmatic airways and correlates with expression of Th2-attracting chemokines and disease severity. J Immunol. 2005; 174: 8183–8190. 15944327 10.4049/jimmunol.174.12.8183

[59] Chai R Liu B Qi F The significance of the levels of IL-4, IL-31 and TLSP in patients with asthma and/or rhinitis. Immunotherapy. 2017; 9: 331–337. 28303765 10.2217/imt-2016-0131

[60] Chauhan A Singh M Agarwal A Paul N Correlation of TSLP, IL-33, and CD4 + CD25 + FOXP3 + T regulatory (Treg) in pediatric asthma. J Asthma. 2015; 52: 868–872. 26287664 10.3109/02770903.2015.1026441

[61] Berraïes A Hamdi B Ammar J Hamzaoui K Hamzaoui A Increased expression of thymic stromal lymphopoietin in induced sputum from asthmatic children. Immunol Lett. 2016; 178: 85–91. 27528425 10.1016/j.imlet.2016.08.004

[62] Glück J Rymarczyk B Kasprzak M Rogala B Increased levels of Interleukin-33 and Thymic Stromal Lymphopoietin in exhaled breath condensate in chronic bronchial asthma. Int Arch Allergy Immunol. 2016; 169: 51–56. 26953567 10.1159/000444017

[63] Li Y Wang W Lv Z Li Y Chen Y Huang K Corrigan CJ Ying S Elevated expression of IL-33 and TSLP in the airways of human asthmatics in vivo: A potential bi-omarker of severe refractory disease. J Immunol. 2018; 200: 2253–2262. 29453280 10.4049/jimmunol.1701455

[64] Bjerregaard A Laing IA Poulsen N Backer V Sverrild A Fally M Khoo SK Barrett L Baltic S Thompson PJ Chidlow G Sikazwe C Smith DW Bochkov YA Le Souëf P Porsbjerg C Characteristics associated with clinical severity and inflammatory phenotype of naturally occurring virus-induced exacerbations of asthma in adults. Respir Med. 2017; 123: 34–41. 28137494 10.1016/j.rmed.2016.12.010 PMC5462105

[65] West EE Kashyap M Leonard WJ TSLP: A key regulator of asthma pathogenesis. Drug Discov Today Dis Mech. 2012; 9: e83–e88. 10.1016/j.ddmec.2012.09.003 PMC3859144 24348685

[66] Hirota T Takahashi A Kubo M Tsunoda T Tomita K Doi S Fujita K Miyatake A Enomoto T Miyagawa T Adachi M Tanaka H Niimi A Matsumoto H Ito I Masuko H Sakamoto T Hizawa N Taniguchi M Lima JJ Genome-wide association study identifies three new susceptibility loci for adult asthma in the Japanese population. Nat Genet. 2011; 43: 893–896. 21804548 10.1038/ng.887 PMC4310726

[67] Moffatt MF Gut IG Demenais F Strachan DP Bouzigon E Heath S von Mutius E Farrall M Lathrop M Cookson WOCM A large-scale, consortium-based genomewide association study of asthma. N Engl J Med. 2010; 363: 1211–1221. 20860503 10.1056/NEJMoa0906312 PMC4260321

[68] Torgerson DG Ampleford EJ Chiu GY Gauderman WJ Gignoux CR Graves PE Himes BE Levin AM Mathias RA Hancock DB Baurley JW Eng C Stern DA Celedón JC Rafaels N Capurso D Conti DV Roth LA Soto-Quiros M Togias A Meta-analysis of genome-wide association studies of asthma in ethnically diverse North American populations. Nat Genet. 2011; 43: 887–892. 21804549 10.1038/ng.888 PMC3445408

[69] He JQ Hallstrand TS Knight D Chan-Yeung M Sandford A Tripp B Zamar D Bossé Y Kozyrskyj AL James A Laprise C Daley D A thymic stromal lymphopoietin gene variant is associated with asthma and airway hyperresponsiveness. J Allergy Clin Immunol. 2009; 124: 222–229. 19539984 10.1016/j.jaci.2009.04.018

[70] Hunninghake GM Soto-Quirós ME Avila L Kim HP Lasky-Su J Rafaels N Ruczinski I Beaty TH Mathias RA Barnes KC Wilk JB O’Connor GT Gauderman WJ Vora H Baurley JW Gilliland F Liang C Sylvia JS Klanderman BJ Sharma SS TSLP polymorphisms are associated with asthma in a sex-specific fashion. Allergy. 2010; 65: 1566–1575. 20560908 10.1111/j.1398-9995.2010.02415.x PMC2970693

[71] Hui CCK Yu A Heroux D Akhabir L Sandford AJ Neighbour H Denburg JA Thymic stromal lymphopoietin (TSLP) secretion from human nasal epithelium is a function of TSLP genotype. Mucosal Immunol. 2015; 8: 993–999. 25515628 10.1038/mi.2014.126

[72] Moorehead A Hanna R Heroux D Neighbour H Sandford A Gauvreau GM Sommer DD Denburg JA Akhabir L A thymic stromal lymphopoietin polymorphism may provide protection from asthma by altering gene expression. Clin Exp Allergy. 2020; 50: 471–478. 31943442 10.1111/cea.13568

[73] Buchheit KM Cahill KN Katz HR Murphy KC Feng C Lee-Sarwar K Lai J Bhattacharyya N Israel E Boyce JA Laidlaw TM Thymic stromal lymphopoietin controls prostaglandin D2 generation in patients with aspirin-exacerbated respiratory disease. J Allergy Clin Immunol. 2016; 137: 1566–1576.e5. 26691435 10.1016/j.jaci.2015.10.020 PMC4860132

[74] Dölle-Bierke S Plank-Habibi S Schäfer C Ahrens B Ballmer-Weber B Beyer K Blümchen K Huttegger I Jappe U Kleine-Tebbe J Lange L Lau S Lepp U Mahler V Müller S Saloga J Schnadt S Szepfalusi Z Treudler R Wassmann-Otto A Dietary impli-cations in acetylsalicylic acid intolerance. Allergo J Int. 2020; 29: 93–96.

[75] Gauvreau GM O’Byrne PM Boulet L-P Wang Y Cockcroft D Bigler J FitzGerald JM Boedigheimer M Davis BE Dias C Gorski KS Smith L Bautista E Comeau MR Leigh R Parnes JR Effects of an anti-TSLP antibody on allergen-induced asthmatic responses. N Engl J Med. 2014; 370: 2102–2110. 24846652 10.1056/NEJMoa1402895

[76] Aun MV Bonamichi-Santos R Arantes-Costa FM Kalil J Giavina-Bianchi P Animal models of asthma: utility and limitations. J Asthma Allergy. 2017; 10: 293–301. 29158683 10.2147/JAA.S121092 PMC5683778

[77] Sagar S Akbarshahi H Uller L Translational value of animal models of asthma: Challenges and promises. Eur J Pharmacol. 2015; 759: 272–277. 25823808 10.1016/j.ejphar.2015.03.037

[78] Eyerich K Eyerich S Th22 cells in allergic disease. Allergo J Int. 2015; 24: 1–7. 26120541 10.1007/s40629-015-0039-3 PMC4479457

[79] Hausmann O Warnatz K Immunodeficiency in adults a practical guide for the allergist. Allergo J Int. 2014; 23: 261–268. 26120536 10.1007/s40629-014-0030-4 PMC4479546

[80] Huber M Lohoff M Change of paradigm: CD8+ T cells as important helper for CD4+ T cells during asthma and autoimmune encephalomyelitis. Allergo J Int. 2015; 24: 8–15. 26120542 10.1007/s40629-015-0038-4 PMC4479451

[81] Ohnmacht C Microbiota, regulatory T cell subsets, and allergic disorders. Allergo J Int. 2016; 25: 114–123. 27656354 10.1007/s40629-016-0118-0 PMC5016534

[82] Ono S Kabashima K Novel insights into the role of immune cells in skin and inducible skin-associated lymphoid tissue (iSALT). Allergo J Int. 2015; 24: 170–179. 27069837 10.1007/s40629-015-0065-1 PMC4792357

[83] Pusch E Renz H Skevaki C Respiratory virus-induced heterologous immunity: Part of the problem or part of the solution? Allergo J Int. 2018; 27: 79–96. 32300267 10.1007/s15007-018-1580-4 PMC7149200

[84] Raap U Sumbayev VV Gibbs BF The role of basophils in allergic inflammation. Allergo J. 2015; 24: 28–33.

[85] Leuppi JD Salome CM Jenkins CR Anderson SD Xuan W Marks GB Koskela H Brannan JD Freed R Andersson M Chan HK Woolcock AJ Predictive markers of asthma exacerbation during stepwise dose reduction of inhaled corticosteroids. Am J Respir Crit Care Med. 2001; 163: 406–412. 11179114 10.1164/ajrccm.163.2.9912091

[86] van Veen IH Ten Brinke A Gauw SA Consistency of sputum eosinophilia in difficult-to-treat asthma: a 5-year follow-up study. J Allergy Clinical Immunol. 2009; 124: 615–617. 19733302 10.1016/j.jaci.2009.06.029

[87] Lemière C Ernst P Olivenstein R Yamauchi Y Govindaraju K Ludwig MS Martin JG Hamid Q Airway inflammation assessed by invasive and noninvasive means in severe asthma: eosinophilic and noneosinophilic phenotypes. J Allergy Clin Immunol. 2006; 118: 1033–1039. 17088126 10.1016/j.jaci.2006.08.003

[88] Cook EB Stahl JL Schwantes EA Fox KE Mathur SK IL-3 and TNFα increase Thymic Stromal Lymphopoietin Receptor (TSLPR) expression on eosinophils and enhance TSLP-stimulated degranulation. Clin Mol Allergy. 2012; 10: 8. 22838633 10.1186/1476-7961-10-8 PMC3418208

[89] Wong CK Hu S Cheung PFY Lam CW Thymic stromal lymphopoietin induces chemotactic and prosurvival effects in eosinophils: implications in allergic inflammation. Am J Respir Cell Mol Biol. 2010; 43: 305–315. 19843704 10.1165/rcmb.2009-0168OC

[90] Morshed M Yousefi S Stöckle C Simon HU Simon D Thymic stromal lymphopoietin stimulates the formation of eosinophil extracellular traps. Allergy. 2012; 67: 1127–1137. 22764833 10.1111/j.1398-9995.2012.02868.x

[91] Hui CCK Rusta-Sallehy S Asher I Heroux D Denburg JA The effects of thymic stromal lymphopoietin and IL-3 on human eosinophil-basophil lineage commitment: Relevance to atopic sensitization. Immun Inflamm Dis. 2014; 2: 44–55. 25400924 10.1002/iid3.20 PMC4220668

[92] Salter BMA Smith SG Mukherjee M Plante S Krisna S Nusca G Oliveria JP Irshad A Gauvreau GM Chakir J Nair P Sehmi R Human bronchial epithelial cell-derived factors from severe asthmatic subjects stimu-late eosinophil differentiation. Am J Respir Cell Mol Biol. 2018; 58: 99–106. 28853918 10.1165/rcmb.2016-0262OC

[93] Smith SG Gugilla A Mukherjee M Merim K Irshad A Tang W Kinoshita T Watson B Oliveria JP Comeau M O’Byrne PM Gauvreau GM Sehmi R Thymic stromal lymphopoietin and IL-33 modulate migration of hematopoietic progenitor cells in patients with allergic asthma. J Allergy Clin Immunol. 2015; 135: 1594–1602. 25656998 10.1016/j.jaci.2014.12.1918

[94] Allakhverdi Z Comeau MR Smith DE Toy D Endam LM Desrosiers M Liu YJ Howie KJ Denburg JA Gauvreau GM Delespesse G CD34+ hemopoietic progenitor cells are potent effectors of allergic inflammation. J Allergy Clin Immunol. 2009; 123: 472–478. 19064280 10.1016/j.jaci.2008.10.022

[95] Bartemes KR Kephart GM Fox SJ Kita H Enhanced innate type 2 immune response in peripheral blood from patients with asthma. J Allergy Clin Immunol. 2014; 134: 671–678.e4. 25171868 10.1016/j.jaci.2014.06.024 PMC4149890

[96] Klose CSN Artis D Innate lymphoid cells as regulators of immunity, inflammation and tissue homeostasis. Nat Immunol. 2016; 17: 765–774. 27328006 10.1038/ni.3489

[97] Walker JA McKenzie ANJ Development and function of group 2 innate lymphoid cells. Curr Opin Immunol. 2013; 25: 148–155. 23562755 10.1016/j.coi.2013.02.010 PMC3776222

[98] Camelo A Rosignoli G Ohne Y Stewart RA Overed-Sayer C Sleeman MA May RD IL-33, IL-25, and TSLP induce a distinct phenotypic and activation profile in human type 2 innate lymphoid cells. Blood Adv. 2017; 1: 577–589. 29296700 10.1182/bloodadvances.2016002352 PMC5728348

[99] Chen R Smith SG Salter B El-Gammal A Oliveria JP Obminski C Watson R O’Byrne PM Gauvreau GM Sehmi R Allergen-induced Increases in sputum levels of group 2 innate lymphoid cells in subjects with asthma. Am J Respir Crit Care Med. 2017; 196: 700–712. 28422515 10.1164/rccm.201612-2427OC

[100] Christianson CA Goplen NP Zafar I Irvin C Good JT Rollins DR Gorentla B Liu W Gorska MM Chu H Martin RJ Alam R Persistence of asthma requires multiple feedback circuits involving type 2 innate lymphoid cells and IL-33. J Allergy Clin Immunol. 2015; 136: 59–68.e14. 25617223 10.1016/j.jaci.2014.11.037 PMC4494983

[101] Smith SG Chen R Kjarsgaard M Huang C Oliveria JP O’Byrne PM Gauvreau GM Boulet LP Lemiere C Martin J Nair P Sehmi R Increased numbers of activated group 2 innate lymphoid cells in the airways of patients with severe asthma and persistent airway eosinophilia. J Allergy Clin Immunol. 2016; 137: 75–86.e8. 26194544 10.1016/j.jaci.2015.05.037

[102] Yu QN Guo YB Li X Li CL Tan WP Fan XL Qin ZL Chen D Wen WP Zheng SG Fu QL ILC2 frequency and activity are inhibited by glucocorticoid treatment via STAT pathway in patients with asthma. Allergy. 2018; 73: 1860–1870. 29542140 10.1111/all.13438 PMC6175310

[103] Liu S Verma M Michalec L Liu W Sripada A Rollins D Good J Ito Y Chu H Gorska MM Martin RJ Alam R Steroid resistance of airway type 2 innate lymphoid cells from patients with severe asthma: The role of thymic stromal lymphopoietin. J Allergy Clin Immunol. 2018; 141: 257–268.e6. 28433687 10.1016/j.jaci.2017.03.032 PMC5650571

[104] Sirohi K Verma M Michalec L et al. Identification of MEK2 and CBX7 as Top Steroid Resistant Genes in Airway ILC2s and Lymphocytes from Asthma.. In:AAAAI/WAO Joint Congress 2018.

[105] Nagarkar DR Poposki JA Comeau MR Biyasheva A Avila PC Schleimer RP Kato A Airway epithelial cells activate TH2 cytokine production in mast cells through IL-1 and thymic stromal lymphopoietin. J Allergy Clin Immunol. 2012; 130: 225–32.e4. 22633328 10.1016/j.jaci.2012.04.019 PMC3387295

[106] Moon P-D Kim H-M Thymic stromal lymphopoietin is expressed and produced by caspase-1/NF-κB pathway in mast cells. Cytokine. 2011; 54: 239–243. 21463955 10.1016/j.cyto.2011.03.007

[107] Okayama Y Okumura S Sagara H Yuki K Sasaki T Watanabe N Fueki M Sugiyama K Takeda K Fukuda T Saito H Ra C FcepsilonRI-mediated thymic stromal lymphopoietin production by interleukin-4-primed human mast cells. Eur Respir J. 2009; 34: 425–435. 19164348 10.1183/09031936.00121008

[108] Allakhverdi Z Comeau MR Jessup HK Delespesse G Thymic stromal lymphopoietin as a mediator of crosstalk between bronchial smooth muscles and mast cells. J Allergy Clin Immunol. 2009; 123: 958–60.e2. 19348931 10.1016/j.jaci.2009.01.059

[109] Hirano R Hasegawa S Hashimoto K Haneda Y Ohsaki A Ichiyama T Human thymic stromal lymphopoietin enhances expression of CD80 in human CD14+ monocytes/macrophages. Inflamm Res. 2011; 60: 605–610. 21274737 10.1007/s00011-011-0310-0

[110] Reche PA Soumelis V Gorman DM Clifford T Liu Mr M Travis M Zurawski SM Johnston J Liu YJ Spits H de Waal Malefyt R Kastelein RA Bazan JF Human thymic stromal lymphopoietin preferentially stimulates myeloid cells. J Immunol. 2001; 167: 336–343. 11418668 10.4049/jimmunol.167.1.336

[111] Allakhverdi Z Comeau MR Armant M Agrawal R Woodfolk JA Sehmi R Howie KJ Gauvreau GM Delespesse G Mast cell-activated bone marrow mesenchymal stromal cells regulate proliferation and lineage commitment of CD34(+) progenitor cells. Front Immunol. 2013; 4: 461. 24381572 10.3389/fimmu.2013.00461 PMC3865761

[112] Siracusa MC Saenz SA Hill DA Kim BS Headley MB Doering TA Wherry EJ Jessup HK Siegel LA Kambayashi T Dudek EC Kubo M Cianferoni A Spergel JM Ziegler SF Comeau MR Artis D TSLP promotes interleukin-3-independent basophil haematopoiesis and type 2 inflammation. Nature. 2011; 477: 229–233. 21841801 10.1038/nature10329 PMC3263308

[113] Agrawal R Wright PW Woodfolk JA Allergen induces dual upregulation of TSLP receptor on circulating basophils and dendritic cells in atopic dermatitis. J Allergy Clin Immunol. 2012; 129:AB69.

[114] Agrawal R Wisniewski J Yu MD Kennedy JL Platts-Mills T Heymann PW Woodfolk JA Infection with human rhinovirus 16 promotes enhanced IgE responsiveness in basophils of atopic asthmatics. Clin Exp Allergy. 2014; 44: 1266–1273. 25113532 10.1111/cea.12390 PMC4191843

[115] Boita M Heffler E Omedè P Bellocchia M Bussolino C Solidoro P Giorgis V Guerrera F Riva G Brussino L Bucca C Rolla G Basophil membrane expression of epithelial cytokine receptors in patients with severe asthma. Int Arch Allergy Immunol. 2018; 175: 171–176. 29402810 10.1159/000486314

[116] Salter BM Oliveria JP Nusca G Smith SG Watson RM Comeau M Sehmi R Gauvreau GM Thymic stromal lymphopoietin activation of basophils in patients with allergic asthma is IL-3 dependent. J Allergy Clin Immunol. 2015; 136: 1636–1644. 25962901 10.1016/j.jaci.2015.03.039

[117] Salter BM Oliveria JP Nusca G Smith SG Tworek D Mitchell PD Watson RM Sehmi R Gauvreau GM IL-25 and IL-33 induce Type 2 inflammation in basophils from subjects with allergic asthma. Respir Res. 2016; 17:5. 26762527 10.1186/s12931-016-0321-z PMC4712475

[118] Hanabuchi S Ito T Park WR Watanabe N Shaw JL Roman E Arima K Wang YH Voo KS Cao W Liu YJ Thymic stromal lymphopoietin-activated plasmacytoid dendritic cells induce the generation of FOXP3+ regulatory T cells in human thymus. J Immunol. 2010; 184: 2999–3007. 20173030 10.4049/jimmunol.0804106 PMC3325785

[119] Arima K Watanabe N Hanabuchi S Chang M Sun SC Liu YJ Distinct signal codes generate dendritic cell functional plasticity. Sci Signal. 2010; 3: ra4. 20086239 10.1126/scisignal.2000567 PMC3325779

[120] Ito T Wang Y-H Duramad O Hori T Delespesse GJ Watanabe N Qin FX Yao Z Cao W Liu YJ TSLP-activated dendritic cells induce an inflammatory T helper type 2 cell response through OX40 ligand. J Exp Med. 2005; 202: 1213–1223. 16275760 10.1084/jem.20051135 PMC2213234

[121] Liu Y-J Thymic stromal lymphopoietin: master switch for allergic inflammation. J Exp Med. 2006; 203: 269–273. 16432252 10.1084/jem.20051745 PMC2118215

[122] Elder MJ Webster SJ Williams DL Gaston JS Goodall JC TSLP production by dendritic cells is modulated by IL-1β and components of the endoplasmic reticulum stress response. Eur J Immunol. 2016; 46: 455–463. 26573878 10.1002/eji.201545537 PMC4783504

[123] Kashyap M Rochman Y Spolski R Samsel L Leonard WJ Thymic stromal lymphopoietin is produced by dendritic cells. J Immunol. 2011; 187: 1207–1211. 21690322 10.4049/jimmunol.1100355 PMC3140600

[124] Watanabe N Hanabuchi S Soumelis V Yuan W Ho S de Waal Malefyt R Liu YJ Human thymic stromal lymphopoietin promotes dendritic cell-mediated CD4+ T cell homeostatic expansion. Nat Immunol. 2004; 5: 426–434. 14991051 10.1038/ni1048

[125] Wang Y-H Angkasekwinai P Lu N Voo KS Arima K Hanabuchi S Hippe A Corrigan CJ Dong C Homey B Yao Z Ying S Huston DP Liu YJ IL-25 augments type 2 immune responses by enhancing the expansion and functions of TSLP-DC-activated Th2 memory cells. J Exp Med. 2007; 204: 1837–1847. 17635955 10.1084/jem.20070406 PMC2118667

[126] Froidure A Shen C Gras D Van Snick J Chanez P Pilette C Myeloid dendritic cells are primed in allergic asthma for thymic stromal lymphopoietin-mediated induction of Th2 and Th9 responses. Allergy. 2014; 69: 1068–1076. 24888572 10.1111/all.12435

[127] Huang L Zhang X Wang M Chen Z Yan Y Gu W Tan J Jiang W Ji W Exosomes from thymic stromal lymphopoietin-activated dendritic cells promote Th2 differentiation through the OX40 ligand. Pathobiology. 2019; 86: 111–117. 30408778 10.1159/000493013

[128] Nguyen KD Vanichsarn C Nadeau KC TSLP directly impairs pulmonary Treg function: association with aberrant tolerogenic immunity in asthmatic airway. Allergy Asthma Clin Immunol. 2010; 6: 4. 20230634 10.1186/1710-1492-6-4 PMC3161393

[129] Wang Y-H Ito T Wang Y-H Homey B Watanabe N Martin R Barnes CJ McIntyre BW Gilliet M Kumar R Yao Z Liu YJ Maintenance and polarization of human TH2 central memory T cells by thymic stromal lymphopoietin-activated dendritic cells. Immunity. 2006; 24: 827–838. 16782037 10.1016/j.immuni.2006.03.019

[130] Rochman I Watanabe N Arima K Liu YJ Leonard WJ Cutting edge: direct action of thymic stromal lymphopoietin on activated human CD4+ T cells. J Immunol. 2007; 178: 6720–6724. 17513717 10.4049/jimmunol.178.11.6720

[131] Rochman Y Dienger-Stambaugh K Richgels PK Lewkowich IP Kartashov AV Barski A Khurana Hershey GK Leonard WJ Singh H TSLP signaling in CD4 T cells programs a pathogenic T helper 2 cell state. Sci Signal. 2018; 11: eaam8858. 29535264 10.1126/scisignal.aam8858 PMC6039124

[132] Akamatsu T Watanabe N Kido M Saga K Tanaka J Kuzushima K Nishio A Chiba T Human TSLP directly enhances expansion of CD8+ T cells. Clin Exp Immunol. 2008; 154: 98–106. 18727630 10.1111/j.1365-2249.2008.03731.x PMC2561086

[133] Jones CE Chan K Interleukin-17 stimulates the expression of interleukin-8, growth-related oncogene-alpha, and granulocyte-colony-stimulating factor by human airway epithelial cells. Am J Respir Cell Mol Biol. 2002; 26: 748–753. 12034575 10.1165/ajrcmb.26.6.4757

[134] Chesné J Braza F Mahay G Brouard S Aronica M Magnan A IL-17 in severe asthma. Where do we stand? Am J Respir Crit Care Med. 2014; 190: 1094–1101. 25162311 10.1164/rccm.201405-0859PP

[135] Tanaka J Watanabe N Kido M Saga K Akamatsu T Nishio A Chiba T Human TSLP and TLR3 ligands promote differentiation of Th17 cells with a central memory phenotype under Th2-polarizing conditions. Clin Exp Allergy. 2009; 39: 89–100. 19055649 10.1111/j.1365-2222.2008.03151.x PMC7164823

[136] Liang Y Yu B Chen J Wu H Xu Y Yang B Lu Q Thymic stromal lymphopoietin epigenetically upregulates Fc receptor γ subunit-related receptors on antigen-presenting cells and induces T 2/T 17 polarization through dectin-2. J Allergy Clin Immunol. 2019; 144: 1025–1035.e7. 31251950 10.1016/j.jaci.2019.06.011

[137] West EE Spolski R Kazemian M Yu ZX Kemper C Leonard WJ A TSLP-complement axis mediates neutrophil killing of methicillin-resistant Staphylococcus aureus. Sci Immunol. 2016; 1: eaaf8471. 28783679 10.1126/sciimmunol.aaf8471 PMC8530006

[138] Wang W Li Y Lv Z Chen Y Li Y Huang K Corrigan CJ Ying S Bronchial allergen challenge of patients with atopic asthma triggers an alarmin (IL-33, TSLP, and IL-25) response in the airways epithelium and submucosa. J Immunol. 2018; 201: 2221–2231. 30185520 10.4049/jimmunol.1800709

[139] Corren J Parnes JR Wang L Mo M Roseti SL Griffiths JM van der Merwe R Tezepelumab in Adults with Uncontrolled Asthma. N Engl J Med. 2017; 377: 936–946. 28877011 10.1056/NEJMoa1704064

[140] Fehrenbach H Wagner C Wegmann M Airway remodeling in asthma: what really matters. Cell Tissue Res. 2017; 367: 551–569. 28190087 10.1007/s00441-016-2566-8 PMC5320023

[141] Nino G Huseni S Perez GF Pancham K Mubeen H Abbasi A Wang J Eng S Colberg-Poley AM Pillai DK Rose MC Directional secretory response of double stranded RNA-induced thymic stromal lymphopoetin (TSLP) and CCL11/eotaxin-1 in human asthmatic airways. PLoS One. 2014; 9: e115398. 25546419 10.1371/journal.pone.0115398 PMC4278901

[142] Redhu NS Gounni AS Function and mechanisms of TSLP/TSLPR complex in asthma and COPD. Clin Exp Allergy. 2012; 42: 994–1005. 22168549 10.1111/j.1365-2222.2011.03919.x

[143] Shan L Redhu NS Saleh A Halayko AJ Chakir J Gounni AS Thymic stromal lymphopoietin receptor-mediated IL-6 and CC/CXC chemokines expression in human airway smooth muscle cells: role of MAPKs (ERK1/2, p38, and JNK) and STAT3 pathways. J Immunol. 2010; 184: 7134–7143. 20483734 10.4049/jimmunol.0902515

[144] Smelter DF Sathish V Thompson MA Pabelick CM Vassallo R Prakash YS Thymic stromal lymphopoietin in cigarette smoke-exposed human airway smooth muscle. J Immunol. 2010; 185: 3035–3040. 20660708 10.4049/jimmunol.1000252 PMC3681514

[145] Futamura K Orihara K Hashimoto N Morita H Fukuda S Sagara H Matsumoto K Tomita Y Saito H Matsuda A beta2-Adrenoceptor agonists enhance cytokine-induced release of thymic stromal lymphopoietin by lung tissue cells. Int Arch Allergy Immunol. 2010; 152: 353–361. 20185927 10.1159/000288288

[146] Redhu NS Saleh A Halayko AJ Ali AS Gounni AS Essential role of NF-κB and AP-1 transcription factors in TNF-α-induced TSLP expression in human airway smooth muscle cells. Am J Physiol Lung Cell Mol Physiol. 2011; 300: L479–L485. 21148792 10.1152/ajplung.00301.2009

[147] Wu J Dong F Wang R-A Wang J Zhao J Yang M Gong W Cui R Dong L Central role of cellular senescence in TSLP-induced airway remodeling in asthma. PLoS One. 2013; 8: e77795. 24167583 10.1371/journal.pone.0077795 PMC3805661

[148] Cao L Liu F Liu Y Liu T Wu J Zhao J Wang J Li S Xu J Dong L TSLP promotes asthmatic airway remodeling via p38-STAT3 signaling pathway in human lung fibroblast. Exp Lung Res. 2018; 44: 288–301. 30428724 10.1080/01902148.2018.1536175

[149] Wu J Liu F Zhao J Wei Y Lv J Dong F Bi W Wang X Wang J Liu W Dong L Tian H Thymic stromal lymphopoietin promotes asthmatic airway remodelling in human lung fibroblast cells through STAT3 signalling pathway. Cell Biochem Funct. 2013; 31: 496–503. 23192865 10.1002/cbf.2926

[150] Li Y Lund C Nervik I Loevenich S Døllner H Anthonsen MW Johnsen IB Characterization of signaling pathways regulating the expression of pro-inflammatory long form thymic stromal lymphopoietin upon human metapneumovirus infection. Sci Rep. 2018; 8: 883. 29343779 10.1038/s41598-018-19225-0 PMC5772477

[151] Wieczfinska J Pawliczak R Thymic stromal lymphopoietin and apocynin alter the expression of airway remodeling factors in human rhinovirus-infected cells. Immunobiology. 2017; 222: 892–899. 28545810 10.1016/j.imbio.2017.05.010

[152] Birben E Sahiner UM Karaaslan C Yavuz TS Cosgun E Kalayci O Sackesen C The genetic variants of thymic stromal lymphopoietin protein in children with asthma and allergic rhinitis. Int Arch Allergy Immunol. 2014; 163: 185–192. 24525665 10.1159/000358488

[153] GlaxoSmithKline. Single ascending doses study of Anti-Interleukin-7 receptor α monoclonal antibody (GSK2618960) in healthy volunteers. In: May 9, 2017 ed. ClinicalTrials.gov 2017.

[154] Ellis J van Maurik A Fortunato L Gisbert S Chen K Schwartz A McHugh S Want A Santos Franco S Oliveira JJ Price J Coles A Brown K Su D Craigen JL Yang J Brett S Davis B Cheriyan J Kousin-Ezewu O Anti-IL-7 receptor α monoclonal antibody (GSK2618960) in healthy subjects – a randomized, double-blind, placebo-controlled study. Br J Clin Pharmacol. 2019; 85: 304–315. 30161291 10.1111/bcp.13748 PMC6339973

[155] Mukherjee M Bulir DC Radford K Kjarsgaard M Huang CM Jacobsen EA Ochkur SI Catuneanu A Lamothe-Kipnes H Mahony J Lee JJ Lacy P Nair PK Sputum autoantibodies in patients with severe eosinophilic asthma. J Allergy Clin Immunol. 2018; 141: 1269–1279. 28751233 10.1016/j.jaci.2017.06.033

[156] Pham TH Ren P Parnes JR Tezepelumab reduces multiple key inflammatory biomarkers in patients with severe, uncontrolled asthma in the Phase 2b PATHWAY Study. Am J Respir Crit Care Med. 2019; 199.

[157] Sridhar S Zhao W Pham T-H ezepelumab decreases matrix remodelling and inflammatory pathways in patients with asthma. In: ERS International Congress 2019 abstracts. Madrid, Spain; 2019.

[158] Novartis Pharmaceuticals A randomized, subject- and investigator-blinded, placebo-controlled, parallel-design, bronchoprovocation study to evaluate the safety, tolerability, pharmacokinetics and pharmacodynamics of multiple doses of inhaled csj117 in adult subjects with mild atopic asthma. In: ClinicalTrials.gov; 2021.

[159] AstraZeneca. Study to evaluate Tezepelumab on airway inflammation in adults with uncontrolled asthma (CASCADE). In. ClinicalTrials.gov 2020.

[160] AstraZeneca. Study to evaluate the efficacy and safety of Tezepelumab in reducing oral corticosteroid use in adults with oral corticosteroid dependent asthma (SOURCE). In: ClinicalTrials.gov; 2021. 10.1186/s12931-020-01503-z PMC7550846 33050928

[161] AstraZeneca. Study to evaluate Tezepelumab on airway inflammation in adults with uncontrolled asthma (CASCADE). In: ClinicalTrials.gov; 2020.

[162] AstraZeneca. Extension study to evaluate the safety and tolerability of Tezepelumab in adults and adolescents with severe, uncontrolled asthma (DESTINATION). In: ClinicalTrials.gov; 2021.

[163] Klimek L Pfaar O Worm M Eiwegger T Hagemann J Ollert M Untersmayr E Hoffmann-Sommergruber K Vultaggio A Agache I Bavbek S Bossios A Casper I Chan S Chatzipetrou A Vogelberg C Firinu D Kauppi P Kolios A Kothari A Anwendung von Biologika bei allergischen und Typ-2-entzündlichen Erkrankungen in der aktuellen Covid-19-Pandemie : Positionspapier des Ärzteverbands Deutscher Allergologen (AeDA)A, der Deutschen Gesellschaft für Allergologie und klinische Immunologie (DGAKI)B, der Gesellschaft für Pädiatrische Allergologie und Umweltmedizin (GPA)C, der Österreichischen Gesellschaft für Allergologie und Immunologie (ÖGAI)D, der Luxemburgischen Gesellschaft für Allergologie und Immunologie (LGAI)E, der Österreichischen Gesellschaft für Pneumologie (ÖGP)F in Kooperation mit der deutschen, österreichischen, und schweizerischen ARIA-GruppeG und der Europäischen Akademie für Allergologie und Klinische Immunologie (EAACI)H. Allergo J. 2020; 29: 14–27. 10.1007/s15007-020-2553-y PMC7289636 32546898

[164] Nagarkar DR Poposki JA Tan BK Comeau MR Peters AT Hulse KE Suh LA Norton J Harris KE Grammer LC Chandra RK Conley DB Kern RC Schleimer RP Kato A Thymic stromal lymphopoietin activity is increased in nasal polyps of patients with chronic rhinosinusitis. J Allergy Clin Immunol. 2013; 132: 593–600.e12. 23688414 10.1016/j.jaci.2013.04.005 PMC3759596

[165] Poposki JA Klingler AI Stevens WW Peters AT Hulse KE Grammer LC Schleimer RP Welch KC Smith SS Sidle DM Conley DB Tan BK Kern RC Kato A Proprotein convertases generate a highly functional heterodimeric form of thymic stromal lymphopoietin in humans. J Allergy Clin Immunol. 2017; 139: 1559–1567.e8. 27744031 10.1016/j.jaci.2016.08.040 PMC5389936

[166] Harada M Hirota T Jodo AI Doi S Kameda M Fujita K Miyatake A Enomoto T Noguchi E Yoshihara S Ebisawa M Saito H Matsumoto K Nakamura Y Ziegler SF Tamari M Functional analysis of the thymic stromal lymphopoietin variants in human bronchial epithelial cells. Am J Respir Cell Mol Biol. 2009; 40: 368–374. 18787178 10.1165/rcmb.2008-0041OC

[167] Varricchi G Pecoraro A Marone G Criscuolo G Spadaro G Genovese A Marone G Thymic stromal lymphopoietin isoforms, inflammatory disorders, and cancer. Front Immunol. 2018; 9: 1595. 30057581 10.3389/fimmu.2018.01595 PMC6053489

[168] Verstraete K Peelman F Braun H Lopez J Van Rompaey D Dansercoer A Vandenberghe I Pauwels K Tavernier J Lambrecht BN Hammad H De Winter H Beyaert R Lippens G Savvides SN Structure and antagonism of the receptor complex mediated by human TSLP in allergy and asthma. Nat Commun. 2017; 8: 14937. 28368013 10.1038/ncomms14937 PMC5382266

[169] Xie Y Takai T Chen X Okumura K Ogawa H Long TSLP transcript expression and release of TSLP induced by TLR ligands and cytokines in human keratinocytes. J Dermatol Sci. 2012; 66: 233–237. 22520928 10.1016/j.jdermsci.2012.03.007

[170] Bjerkan L Schreurs O Engen SA Jahnsen FL Baekkevold ES Blix IJ Schenck K The short form of TSLP is constitutively translated in human keratinocytes and has characteristics of an antimicrobial peptide. Mucosal Immunol. 2015; 8: 49–56. 24850429 10.1038/mi.2014.41

[171] Tsilingiri K Fornasa G Rescigno M Thymic stromal lymphopoietin: to cut a long story short. Cell Mol Gastroenterol Hepatol. 2017; 3: 174–182. 28275684 10.1016/j.jcmgh.2017.01.005 PMC5331833

[172] Fornasa G Tsilingiri K Caprioli F Botti F Mapelli M Meller S Kislat A Homey B Di Sabatino A Sonzogni A Viale G Diaferia G Gori A Longhi R Penna G Rescigno M Dichotomy of short and long thymic stromal lymphopoietin isoforms in inflammatory disorders of the bowel and skin. J Allergy Clin Immunol. 2015; 136: 413–422. 26014813 10.1016/j.jaci.2015.04.011 PMC4534776

[173] Kuroda Y Yuki T Takahashi Y Sakaguchi H Matsunaga K Itagaki H Long form of thymic stromal lymphopoietin of keratinocytes is induced by protein allergens. J Immunotoxicol. 2017; 14: 178–187. 28720058 10.1080/1547691X.2017.1349220

[174] Demehri S Turkoz A Manivasagam S Yockey LJ Turkoz M Kopan R Elevated epidermal thymic stromal lymphopoietin levels establish an antitumor environment in the skin. Cancer Cell. 2012; 22: 494–505. 23079659 10.1016/j.ccr.2012.08.017 PMC3480666

[175] Klimek L Bergmann K-C Brehler R Pfützner W Zuberbier T Hartmann K Jakob T Novak N Ring J Merk H Hamelmann E Ankermann T Schmidt S Untersmayr E Hötzenecker W Jensen-Jarolim E Brockow K Mahler V Worm M Practical handling of allergic reactions to COVID-19 vaccines: A position paper from German and Austrian Allergy Societies AeDA, DGAKI, GPA and ÖGAI. Allergo J Int. 2021; 30: 1–17. 33898162 10.1007/s40629-021-00165-7 PMC8054127

[176] Klimek L Novak N Hamelmann E Werfel T Wagenmann M Taube C Bauer A Merk H Rabe U Jung K Schlenter W Ring J Chaker A Wehrmann W Becker S Mülleneisen N Nemat K Czech W Wrede H Brehler R Severe allergic reactions after COVID-19 vaccination with the Pfizer/BioNTech vaccine in Great Britain and USA: Position statement of the German Allergy Societies: Medical Association of German Allergologists (AeDA), German Society for Allergology and Clinical Immunology (DGAKI) and Society for Pediatric Allergology and Environmental Medicine (GPA). Allergo J Int. 2021; 30: 51–55. 33643776 10.1007/s40629-020-00160-4 PMC7903024

[177] Klimek L Worm M Lange L Beyer K Rietschel E Vogelberg C Schnadt S Stöcker B Brockow K Hagemann J Bieber T Wehrmann W Becker S Freudelsperger L Mülleneisen NK Nemat K Czech W Wrede H Brehler R Fuchs T Management von Anaphylaxie-gefährdeten Patienten während der Covid-19-Pandemie: Ein Positionspapier des Ärzteverbandes Deutscher Allergologen (AeDA)A, der Deutschen Gesellschaft für Allergologie und klinische Immunologie (DGAKI)B, der Gesellschaft für Pädiatrische Allergologie und Umweltmedizin (GPA)C und des Deutschen Allergie- und Asthmabundes (DAAB)D. Allergo J. 2020; 29: 16–26. 10.1007/s15007-020-2618-y PMC7605140 33162681

